# Evolutionary diversification of galactinol synthases in Rosaceae: adaptive roles of galactinol and raffinose during apple bud dormancy

**DOI:** 10.1093/jxb/erx451

**Published:** 2018-01-24

**Authors:** Vítor da Silveira Falavigna, Diogo Denardi Porto, Yohanna Evelyn Miotto, Henrique Pessoa dos Santos, Paulo Ricardo Dias de Oliveira, Márcia Margis-Pinheiro, Giancarlo Pasquali, Luís Fernando Revers

**Affiliations:** 1 Graduate Program in Cell and Molecular Biology, Centro de Biotecnologia, Universidade Federal do Rio Grande do Sul, Porto Alegre, RS, Brazil; 2 Embrapa Semiárido, Petrolina, PE, Brazil; 3 Embrapa Uva e Vinho, Bento Gonçalves, RS, Brazil

**Keywords:** Apple, bud dormancy, galactinol synthase, *Malus* × *domestica*, raffinose family oligosaccharides, tolerance to water deficit, transgenic expression, whole-genome duplication

## Abstract

Galactinol synthase (GolS) is a key enzyme in the biosynthetic pathway of raffinose family oligosaccharides (RFOs), which play roles in carbon storage, signal transduction, and osmoprotection. The present work assessed the evolutionary history of *GolS* genes across the Rosaceae using several bioinformatic tools. Apple (*Malus* × *domestica*) *GolS* genes were transcriptionally characterized during bud dormancy, in parallel with galactinol and raffinose measurements. Additionally, *MdGolS2*, a candidate to regulate seasonal galactinol and RFO content during apple bud dormancy, was functionally characterized in Arabidopsis. Evolutionary analyses revealed that whole genome duplications have driven *GolS* gene evolution and diversification in Rosaceae speciation. The strong purifying selection identified in duplicated *GolS* genes suggests that differential gene expression might define gene function better than protein structure. Interestingly, *MdGolS2* was differentially expressed during bud dormancy, concomitantly with the highest galactinol and raffinose levels. One of the intrinsic adaptive features of bud dormancy is limited availability of free water; therefore, we generated transgenic Arabidopsis plants expressing *MdGolS2*. They showed higher galactinol and raffinose contents and increased tolerance to water deficit. Our results suggest that MdGolS2 is the major GolS responsible for RFO accumulation during apple dormancy, and these carbohydrates help to protect dormant buds against limited water supply.

## Teaser

Galactinol and raffinose act together to protect dormant buds against limited availability of winter water; the apple galactinol synthases MdGolS1 and MdGolS2 are responsible for their seasonal accumulation during dormancy.

## Introduction

Gene and genome duplication events have yielded the raw genetic material for biological evolution, with a major impact on most eukaryotes, including plants (Zhang, 2003). Whole-genome duplication (WGD) has been a common feature during angiosperm speciation and is considered to be a driving force in evolution due to the innovations associated with these events (Jiao *et al.*, 2011). In flowering plants, several rounds of WGD have already been traced, including an ancient event that occurred 200 million years ago (MYA) and was shared even by basal angiosperms such as *Amborella trichopoda* (*Amborella* Genome Project, 2013). In the particular case of eudicots, a triplication event that occurred about 125 MYA has shaped their genomes, and contributed to molecular and phylogenetic diversification (Jiao *et al.*, 2012). Apple (*Malus* × *domestica* Borkh.), one of the most important fruit crops in temperate regions worldwide, belongs to the Rosaceae family and was subject to these duplication events during its evolution. Moreover, apples also underwent a recent WGD event around 50 MYA that is restricted to the Pyreae clade, which also encompasses pear and various other Rosaceae genera (Velasco *et al.*, 2010; Wu *et al.*, 2013; Xiang *et al.*, 2017). These events triggered the emergence of specific subclades of genes, resulting in unique developmental and metabolic traits.

Temperate fruit crops such as apple are subject to alternating growth and rest periods through the year-round development cycle. The rest period or dormancy is an adaptive trait for plant survival under adverse environmental conditions, and a well-adjusted dormancy cycle is crucial for the full achievement of the plant’s genetic potential (Falavigna *et al.*, 2015*b*). This process is characterized by the formation of stress-tolerant specialized bud structures that undergo a developmental program for the protection of meristems (Rohde and Bhalerao, 2007). Recent findings indicated that C-repeat binding transcription factors (CBFs), major integrators of the cold response and freezing tolerance, may also modulate dormancy in apple (Wisniewski *et al.*, 2015; Artlip *et al.*, 2016). Besides genes responsible for the molecular control of dormancy progression, a series of proteins and metabolites were also shown to be recruited for the protection of bud integrity during winter. Dehydrins, sugars, detoxification enzymes, chaperones, protein kinases, and other compounds stand out in the process (Maruyama *et al.*, 2009; Eremina *et al.*, 2016). Supporting these findings, studies carried out during bud dormancy have demonstrated the induction of many cold- and dehydration-related genes that integrate the CBF regulation pathway, with galactinol synthases (GolS; EC 2.4.1.123) being recurrent in several reports (Derory *et al.*, 2006; Santamaría *et al.*, 2011; Liu *et al.*, 2012; Falavigna *et al.*, 2014; Andersen *et al.*, 2017).

GolS proteins integrate a subfamily of the GT8 family of glycosyltransferases that are involved in the synthesis of diverse sugar conjugates with important structural, storage, and signaling roles, as well as acting as an energy source (Sengupta *et al.*, 2012). GolS is a key enzyme catalysing the first committed step in the biosynthesis of raffinose family oligosaccharides (RFOs), being responsible for galactinol formation using UDP-galactose and *myo*-inositol as substrates (Elsayed *et al.*, 2014). Galactinol is a building block used in the synthesis of raffinose, stachyose, and other RFOs. These sugars have been described as accumulating in response to cold, freezing, and water deficit (Sengupta *et al.*, 2015). Moreover, galactinol and RFOs are compatible solutes that play roles in carbon storage, osmotic adjustments, signal transduction, and membrane and protein stabilization, among others (Krasensky and Jonak, 2012; Elsayed *et al.*, 2014; Sengupta *et al.*, 2015). The accumulation of galactinol and RFOs during the last stages of seed development is responsible for protecting the embryo during seed desiccation, being necessary for proper seed germination in pea (Blöchl *et al.*, 2007). Finally, *GolS* induction during bud dormancy progression likely results in the seasonal mobilization of RFOs that, together with several other proteins and metabolites, may lead to bud protection during winter (Santamaría *et al.*, 2011; Unda *et al.*, 2012; Ibáñez *et al.*, 2013; Falavigna *et al.*, 2014; Andersen *et al.*, 2017).

In the present work, we assessed the evolutionary history of *GolS* genes in the Rosaceae family and identified that WGD events have driven the evolution and diversification of these genes in Rosaceae speciation. However, duplicated genes showed limited functional divergence, suggesting that differential gene expression might better define gene function rather than protein structure. Therefore, apple *GolS* gene expression was characterized during bud dormancy and in several developmental stages, and in fact, *MdGolS* genes presented differential transcript accumulation patterns. Given that one of the intrinsic adaptive features of bud dormancy is limited availability of free water, *MdGolS2*, a strong candidate to regulate seasonal galactinol and RFO content during bud dormancy, was functionally characterized in Arabidopsis. The data gathered in this work demonstrate that MdGolS2 is a major contributor to galactinol and RFO accumulation during apple bud dormancy, and that these carbohydrates act in the protection of bud integrity during adverse environmental conditions.

## Materials and methods

### Plant material

Apple samples were harvested in two experimental orchards located in the cities of Vacaria (−28.513777, −50.881465 and 972 m altitude) and Caçador (−26.836971, −50.975246 and 935 m altitude), Southern Brazil. In Vacaria, 3-year-old ‘Gala Baigent’ trees grafted on ‘Marubakaido’ rootstocks with ‘M.9’ as interstock were arranged in three blocks of 10 plants each. Six developmental stages were partitioned in different tissues and organs resulting in 13 samples according to the Fleckinger scale (EPPO, 1984): dormant buds (A stage; 21 July 2009); buds at initial bursting (C stage; 4 September 2009); flower buds and young leaves (E2 stage; 12 September 2009); 10 mm-diameter whole set-fruits and associated leaves (I stage; 28 October 2009); 40 mm-diameter unripe fruits divided into pulp, seed and skin, as well as leaves (J stage; 8 December 2009). Mature fruits of 70 mm diameter were also sampled and partitioned into pulp, seed, and skin (M stage; 2 February 2010). See Falavigna *et al.* (2015*a*) for images of the developmental stages. Samples were immediately frozen in liquid nitrogen in the field and stored at −80 °C until use. In Caçador, 7-year-old ‘Fuji Standard’ trees grafted on M.7 rootstocks were disposed in three sampling blocks of four plants each. Forty closed terminal buds from each plant were sampled at eight time points from January 2009 to February 2010 always at 11.00 h. Samples were immediately frozen in liquid nitrogen in the field and stored at −80 °C until use. Sampling dates and corresponding chilling hours accumulated by these samples are depicted in Supplementary Table S1 at *JXB* online.

### Gene expression analysis

DNA was isolated from 200 mg mature leaves of ‘Gala Baigent’ apple trees using modified protocols adapted to 2 ml tubes (Lodhi *et al.*, 1994; Lefort and Douglas, 1999). Total RNA was isolated as described in Zeng & Yang (2002) and Falavigna *et al.* (2014), and DNase-treated using the TURBO DNA-free Kit (Ambion, Austin, TX, USA). The GeneAmp RNA PCR Core Kit (Thermo Fisher Scientific, Waltham, MA, USA) was used for cDNA synthesis according to manufacturer’s instructions. DNA-free RNA samples from mature seeds and dormant buds were also employed in 5′ and 3′ rapid amplification of cDNA ends (RACE) reactions using the SMARTer RACE cDNA Amplification Kit (Clontech, Mountain View, CA, USA) according to the manufacturer’s protocol. RACE products were sequenced at ACTGene Ltd (Porto Alegre/RS, Brazil) using an automatic ABI-PRISM 3100 Genetic Analyzer and associated chemistry (Thermo Fisher Scientific). Real-time PCR was performed as described (Falavigna *et al.*, 2015*a*). PCR efficiency and mean relative gene expression were calculated using LinRegPCR v.2012.0 (Ruijter *et al.*, 2009) and the 2^−ΔΔ*C*t^ method (Livak and Schmittgen, 2001), respectively. *ARC5* (accumulation and replication of chloroplast 5), *MDH* (malate dehydrogenase) and *WD40* (transcription factor WD40-like repeat domain) or *ARC5*, *MDH* and *TMp1* (type 1 membrane protein-like) were used as reference genes for closed terminal buds or for organ/tissue samples, respectively (Perini *et al.*, 2014).

### Carbohydrate analysis

Carbohydrates were extracted using protocols modified from Amaral *et al.* (2007) and Eyles *et al.* (2013). Briefly, galactinol, raffinose and sucrose levels were determined from 100 mg pulverized closed terminal buds from ‘Fuji Standard’. Soluble carbohydrates were extracted with 1 ml 80% ethanol in an 80 °C water bath for 30 min. After centrifugation (10 000 *g* for 5 min), the supernatant was stored and samples were re-extracted five times. Three independent extractions were conducted for each biological replicate. In order to measure extraction efficiency, an additional independent extraction was carried out adding 100 mg l^−1^ of each analyte as internal standards. After extraction, a purification step was performed to remove lesser polar compounds. Volumes of 500 μl of chloroform and 400 μl of water were added to 1 ml of total supernatant followed by vigorous shaking. After centrifugation (13 000 *g* for 5 min), supernatant was collected and filtered (0.22 μm). An UPLC (Waters Acquity UPLC system, Milford, MA, USA) coupled to a triple quadrupole mass spectrometer (MS Waters Xevo TQ) was used for separation and quantification of carbohydrates. The chromatographic separation was performed using a combination of a pre-column (2.1 × 5.0 mm, 1.7 µm particles) and a column (2.1 × 100 mm, 1.7 µm particles) (Acquity UPLC BEH amide). The mobile phase consisted of (A) 0.05% ammonia in water and (B) 0.05% ammonia in acetonitrile. A linear gradient condition from 20% A–80% B to 35% A–65% B at 8 min was followed by the initial condition for an additional 7 min. The column was held at 35 °C and the sample compartment at 7 °C. An injection volume of 2 μl was employed and the flow rate was fixed at 0.3 ml min^−1^. Under these conditions, retention times were: sucrose, 4.3 min; raffinose, 6.8 min; and galactinol, 7.4 min. MS was operated in negative ion electrospray mode with 2.5 kV capillary voltage, 150 °C ion source temperature, 800 l h^−1^ desolvation gas (nitrogen) flow, 20 l h^−1^ cone gas flow and 600 °C desolvation temperature. Data acquisition was performed with the MassLynx version 4.1 (Waters) software using the multiple reaction monitoring (MRM) modes. The MRM transitions, cone and collision voltages were, respectively: sucrose, 341→178.4 *m*/*z*, 35 V and 15 V; raffinose 504→221 *m*/*z*, 35 V and 25 V; galactinol 341→179, 33 V and 25 V. The quantification was performed by comparison of the signals to calibration curves from eleven concentrations ranging from 0 to 50 mg l^−1^ of each sugar. Repeated measure ANOVA followed by Tukey’s *post hoc* test with 99% confidence interval was used to evaluate the statistical significance of differences in carbohydrate levels between sampling points using Prism 5.0a (GraphPad Software, La Jolla, CA, USA). To investigate the correlation between *MdGolS* transcriptional level and carbohydrate content, Pearson’s correlation coefficient analysis at a 0.01 significance level was conducted using GraphPad Prism.

### Arabidopsis transformation

With the aim of generating transgenic plants expressing *MdGolS2*, *MdGolS2*’s complete coding sequence was amplified with high fidelity enzymes (Thermo Fisher Scientific) using gene-specific primers (Supplementary Table S2) and cloned into pENTR^™^ Directional TOPO^®^ (Thermo Fisher Scientific). This construction was used to clone its coding DNA sequence (CDS) into the pH7WG2D.1 vector under the CaMV 35S promoter (Karimi *et al.*, 2007) using Gateway^®^ LR Clonase^™^ II Enzyme Mix (Thermo Fisher Scientific) according to the manufacturer’s instructions. The resulting vector was confirmed by sequencing and transformed into Arabidopsis Col-0 plants using the *Agrobacterium tumefaciens* (EHA105) floral dip method (Clough and Bent, 1998). Transformed seeds were sown in plastic pots and 3-week-old plants were evaluated for green fluorescent protein (GFP) fluorescence resulting from the reporter *EGFP* gene present in the vector (Karimi *et al.*, 2007), using a Leica M165FC stereomicroscope. Plants without GFP fluorescence were discarded. Total RNA was isolated from leaves, and real-time PCR was performed using *MdGolS2* gene-specific primers as previously described. The amplicon obtained from each line was sequenced to confirm its transgenic nature. Plants without the transgene were also discarded. Five independent lines were randomly chosen for further analysis. *AtAct2* (actin 2) and *AtCOP1* (constitutive photomorphogenic 1) were employed as reference genes (Supplementary Table S2). Galactinol and raffinose levels were determined as previously described. Student’s *t*-test was used to evaluate the statistical significance of differences in carbohydrate levels between transgenic lines and wild-type plants.

### Water withhold assay

A preliminary water deficit assay was conducted to identify the number of days needed to cause lethal damage in wild-type Arabidopsis plants under our growth conditions. Prior to the water withhold assay, all pots have had their pot capacity attained and had their weight adjusted to approximately 160 g (soil, plant, pot and water). Two-month-old transformed and non-transformed plants were transferred to vats without water for 5 weeks. After treatment, plants were rehydrated for 10 days prior to evaluation. Pots were weighed once a week to measure water loss. Photographs were taken for visual analysis.

### *In silico* analysis

In order to identify predicted gene models coding for GolS in the apple doubled-haploid genome version 1.1 (Daccord *et al.*, 2017), default parameters were employed in Blastp searches using a sequence previously identified by our group as query (ABD15 in Falavigna *et al.*, 2014; Altschul *et al.*, 1990). Resulting sequences were annotated by comparison with the NCBI non-redundant protein database using Blast2GO version 4.1.0 (*E*-value cutoff of 1e^−5^; Conesa *et al.*, 2005) and then screened for the presence of the GT8 domain (PF01501) in the SMART database (Letunic *et al.*, 2012). Primers were designed using Primer3 v0.4.0 (Supplementary Table S2; Rozen & Skaletsky, 2000). The exon/intron structures of *MdGolS* genes were compiled into a figure using the Gene Structure Display Server 2.0 (Hu *et al.*, 2015).

The available annotated genomes from Rosaceous species in GigaDB (http://gigadb.org/), Phytozome (http://phytozome.jgi.doe.gov/pz/portal.html), NCBI (http://www.ncbi.nlm.nih.gov/) and GDR (http://rosaceae.org/) databases were screened for the presence of GolS proteins using the same strategy described above (accessed in June 2017). All identified sequences were trimmed as previously described. Resulting GolS amino acid sequences from apple, black raspberry, Chinese white pear, European pear, Japanese apricot, peach, strawberry, and woodland strawberry (Supplementary Table S3) were aligned using MUSCLE (Edgar, 2004) and phylogenetic relationships were inferred using MrBayes version 3.2.6 (Ronquist *et al.*, 2012). The mixed amino acid substitution model was used in the default settings and 150 thousand generations were run, sampled every 100 generations, with the first 25% of trees discarded as burn-in. The remaining ones were summarized in a consensus tree that was visualized and edited in FigTree version 1.4.2 (http://tree.bio.ed.ac.uk/software/figtree/).

Collinear block analyses were performed by intra- and interspecific genome-wide protein sequence comparison for Rosaceous species using Blastp (*E*-value<1e^−10^; Altschul *et al.*, 1990). Results and gene positions were used as inputs to determine collinear blocks using MCScanX (Wang *et al.*, 2012). The non-synonymous substitution rate (*K*_a_) and synonymous substitution rate (*K*_s_) of paralog genes were calculated using downstream analysis tools implemented in the MCScanX software. *K*_s_ dating was calculated from collinear blocks containing paralogous *GolS* genes. To this purpose, six genes flanking each *GolS* paralog were used. If fewer than 12 homologous genes were found, then all available genes were used instead. The type of gene duplication event was determined using the duplicate_gene_classifier algorithm implemented in MCScanX (Wang *et al.*, 2012).

## Results

### *GolS* genes in apple

The apple genome was screened for GolS sequences by means of Blastp searches. All resulting sequences were compared with SMART and NCBI non-redundant protein databases, and the ones lacking the GT8 domain (PF01501) or not annotated as GolS-like, respectively, were excluded. This led to the identification of eight predicted gene models coding for GolS in apple (Table 1). However, only five of them were successfully amplified when using a pool of cDNAs from 13 different apple tissues and organs (see Supplementary Fig. S1). These five genes were named *MdGolS1*–*5*, and were amplified by RACE and sequenced (Table 1). *MdGolS1*, *2*, *3*, and *4* cDNA sequences showed differences in comparison with the genomic sequences: one nucleotide mismatch without amino acid change, a misprediction in the second exon that resulted in 27 additional nucleotides, three nucleotide mismatches leading to two amino acid changes, and 17 mismatches leading to four amino acid changes, respectively (Supplementary Fig. S2).

**Table 1.** Identification of *GolS* genes in apple

Gene name	Genome accession	GenBank accession	Chromosomal location	Protein size (aa)
*MdGolS1*	MD04G1190900	KY475539	Chr4: 28069630–28071554	333
*MdGolS2*	MD13G1093700	KY475540	Chr13: 6602655–6604528	337
*MdGolS3*	MD13G1147700	KY475541	Chr13: 11543866–11546147	328
*MdGolS4*	MD17G1280400	KY475542	Chr17: 34045432–34047056	348
*MdGolS5*	MD16G1095000	KY475543	Chr16: 6573774–6575140	302
*MdGolS6*	MD09G1288100	—	Chr9: 36789422–36792275	363
*MdGolS7*	MD16G1147600	—	Chr16: 11523700–11526050	298
*MdGolS8*	MD11G1070000	—	Chr11: 5983858–5985447	359

Genome and GenBank accession codes are provided by the ‘The Apple Genome and Epigenome’ (https://iris.angers.inra.fr/gddh13/) and NCBI (http://www.ncbi.nlm.nih.gov/) databases, respectively.

**Fig. 1.**
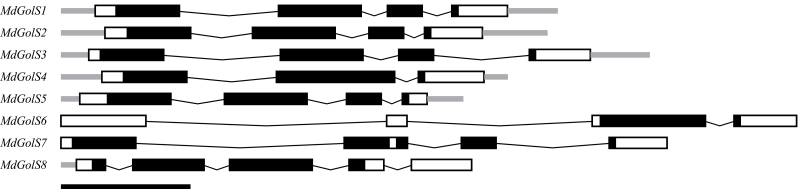
Gene structure of *MdGolS* genes. The GT8-coding domain is colored black and the remaining exon sequences are shown as white boxes. Gray boxes represent untranslated regions, while lines represent introns. Scale bar: 500 bp.

The structures of *MdGolS* genes were retrieved from the apple genome and curated with our sequencing data, when available (Fig. 1). All *MdGolS* genes presented four exons, except *MdGolS4* and *MdGolS8* with three and five exons, respectively. All sequences contained one GT8 domain, except MdGolS7 that presented two. However, both domains in MdGolS7 were truncated. MdGolS6 also presented a truncated version of the GT8 domain.

### GolS evolution among Rosaceae

GolS has already been characterized in a wide phylogenetic scope from green algae to plants (Yin *et al.*, 2010; Sengupta *et al.*, 2012). In this work, a deeper characterization was performed focusing on the Rosaceae family, which integrates the Rosales order and includes important commercial fruit species (Xiang *et al.*, 2017). After an initial screening for the presence of *GolS* genes in the available Rosaceous genomes, retrieved sequences were trimmed, resulting in 52 genes putatively coding for GolS (Supplementary Table S3). Except for strawberry, which is octoploid, all species herein analysed are diploids. The highest number of GolS members was identified in apple and European pear, with eight and 12 members, respectively. Strawberry presented seven members, while black raspberry, Chinese pear, Japanese apricot, peach, and woodland strawberry displayed five members each. The number of *GolS* genes in Chinese pear should be analysed with caution, given that apple and European pear, species evolutionarily close to Chinese pear, presented a higher number of members.

**Fig. 2.**
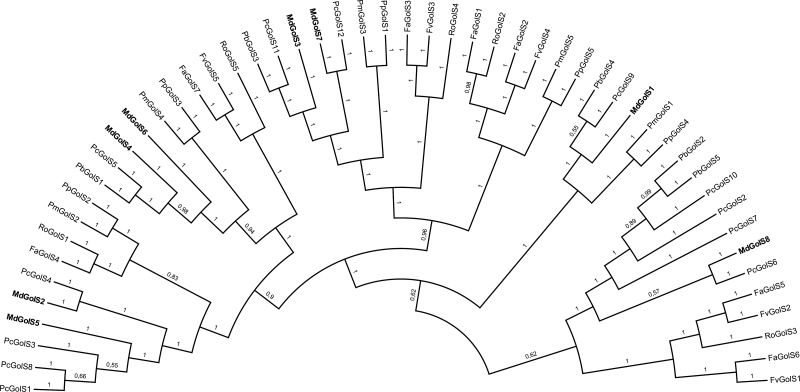
Phylogenetic relationships of GolS protein sequences from eight Rosaceous species. The tree was inferred using MrBayes version 3.2.6 (Ronquist *et al.*, 2012). Branch support is given by *a posteriori* probability value shown next to the corresponding branches (when *>*0.5). Accession codes used in the phylogenetic analysis are depicted in Table S3. Fa, *Fragaria* × *ananassa*; Fv, *Fragaria vesca*; Md, *Malus* × *domestica*; Pb, *Pyrus bretschneideri*; Pc, *Pyrus communis*; Pm, *Prunus mume*; Pp, *Prunus persica*; Ro, *Rubus occidentalis*.

A phylogenetic tree was constructed aiming to identify evolutionary relationships of GolS proteins within the Rosaceae family (Fig. 2). No apple paralogs were identified, and apple orthologs were only identified along with European pear sequences: MdGolS2 with PcGolS4, MdGolS7 with PcGolS12, and MdGolS8 with PcGolS6. MdGolS1 formed an independent cluster with European and Chinese pear orthologs. A similar result was observed for MdGolS3 and MdGolS4. MdGolS5 was present in a cluster with European pear paralogs, while MdGolS6 was present in a cluster with GolS sequences from apple, Chinese and European pear. Japanese apricot and peach sequences grouped as orthologs. The same trend was observed for sequences from black raspberry, strawberry, and woodland strawberry.

In order to trace the *GolS* evolutionary history within the Rosaceae family, collinearity and *K*_s_-dating analyses were performed. Our strategy was based on the comparison of the apple genome with those from black raspberry, Chinese pear, European pear, Japanese apricot, peach, strawberry, and woodland strawberry. These comparisons rendered several collinear regions containing *GolS* genes, allowing us to identify several paralogous pairs (Table 2) as well as several orthologs of *MdGolS* genes (Table 3). A graphical representation of these findings is present in Supplementary Fig. S3. Within the apple genome, eight collinear blocks containing *GolS* genes were identified, between chromosomes 4 and 9 (*MdGolS1*/*MdGolS6*), 4 and 17 (*MdGolS1*/*MdGolS4*), 9 and 16 (*MdGolS5*/*MdGolS6* and *MdGolS6*/*MdGolS7*), 9 and 17 (*MdGolS4*/*MdGolS6*), 13 and 16 (*MdGolS2*/*MdGolS5* and *MdGolS3*/*MdGolS7*), and 16 and 17 (*MdGolS4*/*MdGolS7*). Some of these blocks are syntenic, given that they share the same Pyreae ancestors such as Mdchr9 and 17 and Mdchr13 and 16 (Velasco *et al.*, 2010; Daccord *et al.*, 2017). *GolS* paralogs were also identified for sequences from European pear, Japanese apricot, peach, and woodland strawberry (Table 2). Although no collinear blocks containing *GolS* genes were found between Chinese pear chromosomes, eight blocks were identified linking this genome to apple (Supplementary Fig. S3A; Table 3). Despite the European pear genome not being assembled on chromosomes, the comparison with apple rendered the highest number of collinear blocks (12), with each apple *MdGolS* gene having at least one counterpart in European pear (Supplementary Fig. S3B; Table 3). Japanese apricot and peach showed ten collinear blocks each with apple, besides several collinear regions containing *GolS* genes within each genome (Supplementary Fig. S3C, D; Tables 2 and 3). Finally, the collinearity comparison between apple and woodland strawberry rendered seven collinear blocks between these species (Supplementary Fig. S3E; Table 3). It is worth mentioning that the black raspberry and strawberry genomes are not assembled in chromosomes, which resulted in the presence of few collinear blocks for these species.

**Table 2.** *K*_s_-dating and *K*_a_/*K*_s_ ratios of *GolS* paralogs from different Rosaceous species

Duplicated genes	*K*_s_ dating of the collinear block	*K*_a_/*K*_s_ ratio	Duplicated gene origin
*FvGolS3*/*FvGolS5*	1.12	0.36	WGD
*MdGolS1*/*MdGolS4*	1.53	0.13	WGD
*MdGolS1*/*MdGolS6*	1.54	0.16	WGD
*MdGolS2*/*MdGolS5*	0.25	0.17	WGD
*MdGolS3*/*MdGolS7*	0.21	0.41	WGD
*MdGolS4*/*MdGolS6*	0.15	0.25	WGD
*MdGolS4*/*MdGolS7*	1.37	—	WGD
*MdGolS5*/*MdGolS6*	1.98	0.19	WGD
*MdGolS6*/*MdGolS7*	1.73	0.10	WGD
*PcGolS1*/*PcGolS4*	0.18	0.14	WGD
*PcGolS11*/*PcGolS12*	0.18	0.32	WGD
*PmGolS1*/*PmGolS3*	1.16	0.10	WGD
*PmGolS2*/*PmGolS3*	1.46	0.14	WGD
*PmGolS2*/*PmGolS4*	1.57	—	WGD
*PmGolS3*/*PmGolS5*	1.56	0.15	WGD
*PpGolS1*/*PpGolS4*	1.15	0.12	WGD
*PpGolS2*/*PpGolS3*	1.80	0.05	WGD

*Fv*, *Fragaria vesca*; *K*_a_, non-synonymous substitution rate; *K*_s_, synonymous substitution rate; *Md*,*Malus* × *domestica*; *Pc*, *Pyruscommunis*; *Pm*, *Prunus mume*; *Pp*, *Prunus persica*; WGD, whole-genome duplication.

**Table 3.** List of collinear blocks among six Rosaceous genomes containing orthologs of *MdGolS*genes

Apple	Chinese pear	European pear	Japanese apricot	Peach	Woodland strawberry
*MdGolS1*	*PbGolS4*	*PcGolS9*	*PmGolS1*	*PpGolS4*	*FvGolS1*
*MdGolS2*	—	*PcGolS1*	*PmGolS2*	*PpGolS2*	—
	*PcGolS4*				
*MdGolS3*	*PbGolS3*	*PcGolS11*	*PmGolS1*	*PpGolS1*	*FvGolS1*
	*PcGolS12*	*PmGolS3*	*PpGolS4*	*FvGolS3*	
*MdGolS4*	*PbGolS1*	*PcGolS5*	*PmGolS4*	*PpGolS3*	*FvGolS5*
	*PbGolS4*				
*MdGolS5*	—	*PcGolS1*	*PmGolS2*	*PpGolS2*	—
		*PcGolS4*			
*MdGolS6*	*PbGolS1*	*PcGolS5*	*PmGolS4*	*PpGolS3*	*FvGolS1*
	*PbGolS4*				*FvGolS5*
*MdGolS7*	*PbGolS1*	*PcGolS11*	*PmGolS1*	*PpGolS1*	*FvGolS3*
	*PbGolS3*	*PcGolS12*	*PmGolS3*	*PpGolS3*	
			*PmGolS4*	*PpGolS4*	
*MdGolS8*	—	*PcGolS6*	—	—	—

A schematic representation of the collinear block analysis is present in the Supplementary Figure S3.

The *K*_s_-dating analysis aimed to estimate the time of gene duplications for all collinear blocks containing paralogous *GolS* genes in the Rosaceae family (Table 2). All Rosaceous paralogs had their duplicated gene origin annotated as WGD, suggesting that segmental duplications were the main contributory force to the expansion of *GolS* genes in these genomes. Only apple and European pear paralogs showed *K*_s_ values lower than 0.25, which may correspond to the recent WGD. All *GolS* paralogs from Japanese apricot, peach, and woodland strawberry showed *K*_s_ values ranging from 1.1 to 1.8. The other five apple paralogs, *MdGolS1*/*MdGolS4*, *MdGolS1*/*MdGolS6*, *MdGolS4*/*MdGolS7*, *MdGolS5*/*MdGolS6*, and *MdGolS6*/*MdGolS7*, also presented *K*_s_ values higher than 1. In order to identify if *K*_s_ values higher than 1 corresponded to the triplication event common to all eudicots, *GolS* genes were identified in the grapevine genome, followed by collinearity and *K*_s_-dating analyses. The grapevine genome is a powerful tool for this kind of comparison considering that it has only undergone the triplication event, without subsequent polyploidies (Jaillon *et al.*, 2007; Tang *et al.*, 2008). Twelve *GolS* genes were identified in grapevine, with six collinear blocks containing *GolS* genes that originated in WGD events. The *K*_s_ dating of these collinear blocks ranged from 1 to 1.7 (Supplementary Table S4). However, a region of 41 kbp in the grapevine chromosome 14 presented six grapevine *GolS* genes arranged in tandem. This suggests that tandem duplications can also be held accountable for the number of *GolS* genes in grapevine, although this kind of duplication event is not present in any Rosaceous species.

Finally, the *K*_a_/*K*_s_ ratio was calculated for all Rosaceous *GolS* paralogs. This ratio is used to identify positive selection, which is indicated by values higher than 1. A *K*_a_/*K*_s_ ratio lower than 1 indicates negative selection, whereas a ratio equal to 1 indicates neutral selection. All *K*_a_/*K*_s_ ratios were lower than 0.41 (Table 2), suggesting that these genes experienced purifying selection pressures with limited functional divergence after WGDs.

### Expression patterns of *MdGolS* genes in apple tissues and organs

**Fig. 3.**
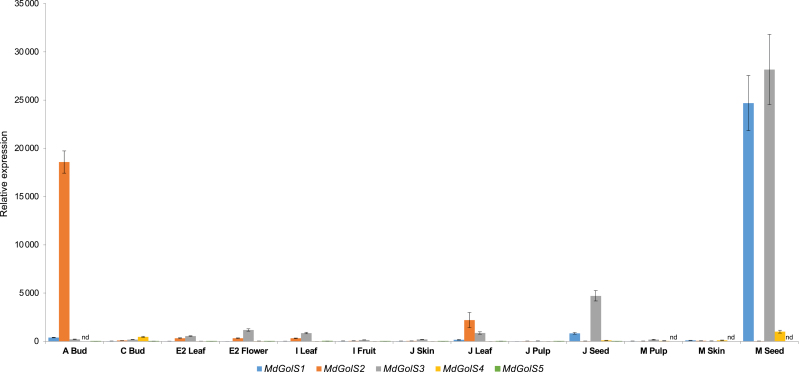
Transcript accumulation kinetics of *MdGolS1*–*5* in 13 apple tissues and organs. Stages A, C, E2, I, and J were sampled according to the Fleckinger phenological scale (EPPO, 1984); ‘M’ stands for mature fruits. nd, not-detected. Gene expression was plotted relative to *MdGolS5* transcript levels in skin from J stage. Standard error bars are shown. (This figure is available in color at *JXB* online.)

Transcript level analysis of *MdGolS* genes was conducted via real-time PCR in 13 apple tissues and organs during the progression of bud dormancy, flowering and fruit ripening. Interestingly, *MdGolS* genes showed remarkable differential expression patterns among the analysed samples. *MdGolS1*, *3*, and *4* were mainly expressed in mature seeds, while *MdGolS2* showed higher mRNA concentrations in dormant buds in comparison with other samples (Fig. 3). *MdGolS1*, *2*, and *3* also presented the highest transcript levels among all samplings and genes analysed. *MdGolS5* showed higher transcript levels in buds at initial bursting and leaves from I and J stages, although it presented the lowest transcript levels over samples in comparison with other *MdGolS* genes. Additionally, *MdGolS4* transcripts were not detected in dormant buds, and *MdGolS5* could not be detected in mature fruits. No transcripts were detected for *MdGolS6*–*8* in any of the tissues or organs analysed (Supplementary Fig. S1).

### *MdGolS* gene expression and sugar content analyses in apple terminal buds during an annual growth and dormancy cycle

**Fig. 4.**
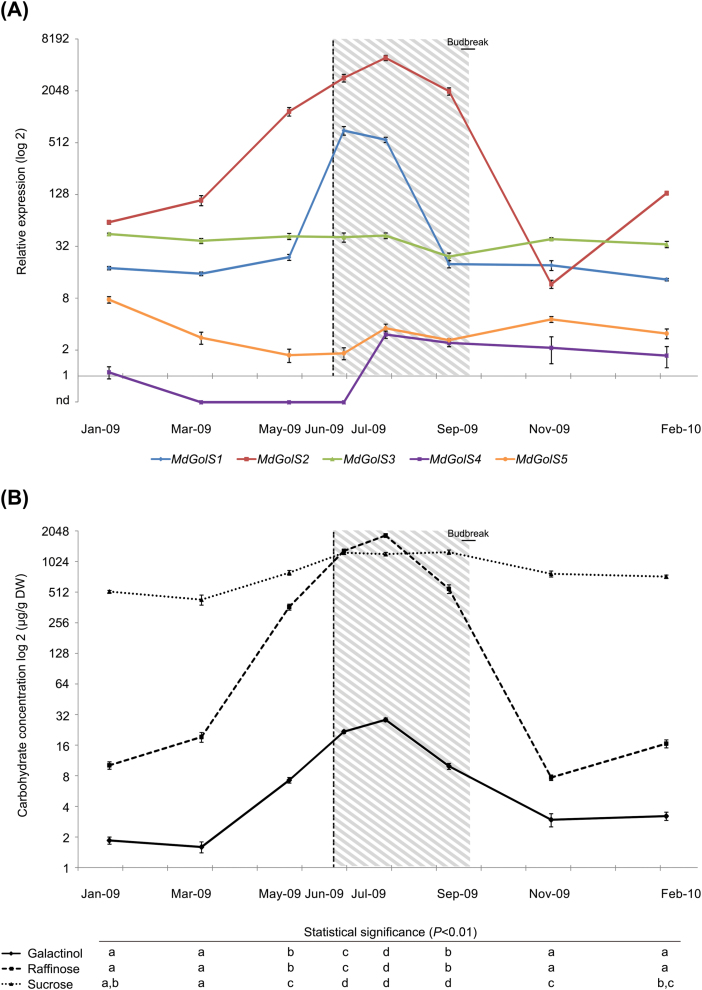
*MdGolS* gene expression and sugar content analysis during an annual growth and dormancy cycle of ‘Fuji Standard’ closed terminal buds. The vertical dashed line indicates the approximate winter solstice (21 June); the shaded area corresponds to the winter season; and the horizontal bar corresponds to the budbreak interval (50% of buds in green tip stage). Standard error bars are shown. (A) Seasonal *MdGolS* transcript level analysis by real-time PCR. Gene expression was plotted relative to *MdGolS4* transcript levels in January 2009. nd, not-detected. (B) Galactinol, raffinose and sucrose levels in apple buds determined by UPLC. Means with the same letter are not significantly different for *P*<0.01 by repeated measure ANOVA followed by Tukey’s test. (This figure is available in color at *JXB* online.)

Closed terminal buds from field-grown ‘Fuji Standard’ apple trees were sampled during a complete annual growth and dormancy cycle from summer 2009 to summer 2010 in southern Brazil. Chilling exposure, and growth cessation and resumption (50% of buds in green tip stage) were followed in order to determine dormancy establishment, fulfillment and release (Supplementary Table S1). The gene expression of *MdGolS1*–*5* was quantified by real-time PCR throughout these samplings (Fig. 4A). *MdGolS1* and *2* displayed a seasonal transcript accumulation during dormancy, with higher amounts in winter buds. Additionally, both genes were down-regulated near budbreak (15 September), with lower transcript levels during spring and summer. It is worth mentioning that *MdGolS2* was 8-fold more expressed during winter than *MdGolS1*. *MdGolS2* reached the highest expression level of all *MdGolS* genes in mid-winter (30 July). Contrastingly, *MdGolS3* and *5* presented low transcriptional variation along the year. Finally, *MdGolS4* expression was completely abolished during dormancy entrance (27 May, autumn) and maintenance (30 June, winter), restoring its transcriptional levels during mid-winter (30 July), spring, and summer.

The seasonal expression profile observed for some *MdGolS* genes prompted us to quantify carbohydrate levels during the same annual sampling cycle of ‘Fuji Standard’ apple buds (Fig. 4B). The sugar extraction efficiency was above 95% for all three analytes. Galactinol and raffinose started to accumulate during dormancy establishment (May, autumn), reaching their highest amounts during deep dormancy (June and July, winter), and restoring low levels after dormancy completion (November 2009 to February 2010, spring and summer). Raffinose levels were approximately 180-fold higher in samples harvested in July in comparison with summer samples, while galactinol levels were approximately 9-fold higher in the same comparison. Moreover, the transcript level of *MdGolS1* and *MdGolS2* along with galactinol and raffinose content during the year showed a strong positive correlation (Table 4). Finally, sucrose levels were higher during winter (June, July, and September) in comparison to autumn, spring and summer samples. A three-fold difference was observed in sucrose levels in the most contrasting points, September and March 2009.

**Table 4.** Pearson’s correlation coefficient between *MdGolS* transcript levels and carbohydrate contents in apple buds of ‘Fuji Standard’

	*MdGolS1*	*MdGolS2*	*MdGolS3*	*MdGolS4*	*MdGolS5*	Galactinol	Raffinose	Sucrose
*MdGolS1*								
*MdGolS2*	0.81	1.00						
*MdGolS3*	0.35	0.10	1.00					
*MdGolS4*	0.67	0.69	−0.24	1.00				
*MdGolS5*	−0.27	−0.32	0.60	−0.68	1.00			
Galactinol	0.91	0.98	0.19	0.72	−0.33	1.00		
Raffinose	0.90	0.99	0.20	0.69	−0.31	1.00	1.00	
Sucrose	0.67	0.82	−0.29	0.71	−0.47	0.81	0.80	1.00

Results from the quantification of *MdGolS1*–*5* and sugar content during eight time points along the year (Fig. 4). Values in bold represent positive correlation between parameters and are significant at the 0.01 level.

### Water deficit assay of transgenic Arabidopsis plants expressing *MdGolS2*

The seasonal accumulation of *MdGolS2* transcripts, combined with the accumulation of galactinol and raffinose during winter, suggests that these metabolites may play important roles during dormancy. Their probable function may be related to increased dormant bud tolerance to dehydration, given that one of the characteristic features of the dormancy process is limited availability of free water (de Faÿ *et al.*, 2000; Améglio *et al.*, 2002; Kalcsits *et al.*, 2009). Within this context, to gain insights about *MdGolS2* function, Arabidopsis plants expressing this gene were generated and exposed to a water deficit assay. This assay aimed to demonstrate the functionality of the MdGolS2 enzyme *in vivo* as well as to test if galactinol and RFOs are able to protect tissues exposed to limited water amounts, mimicking a situation that occurs in apple buds during dormancy (Kalcsits *et al.*, 2009).

**Fig. 5.**
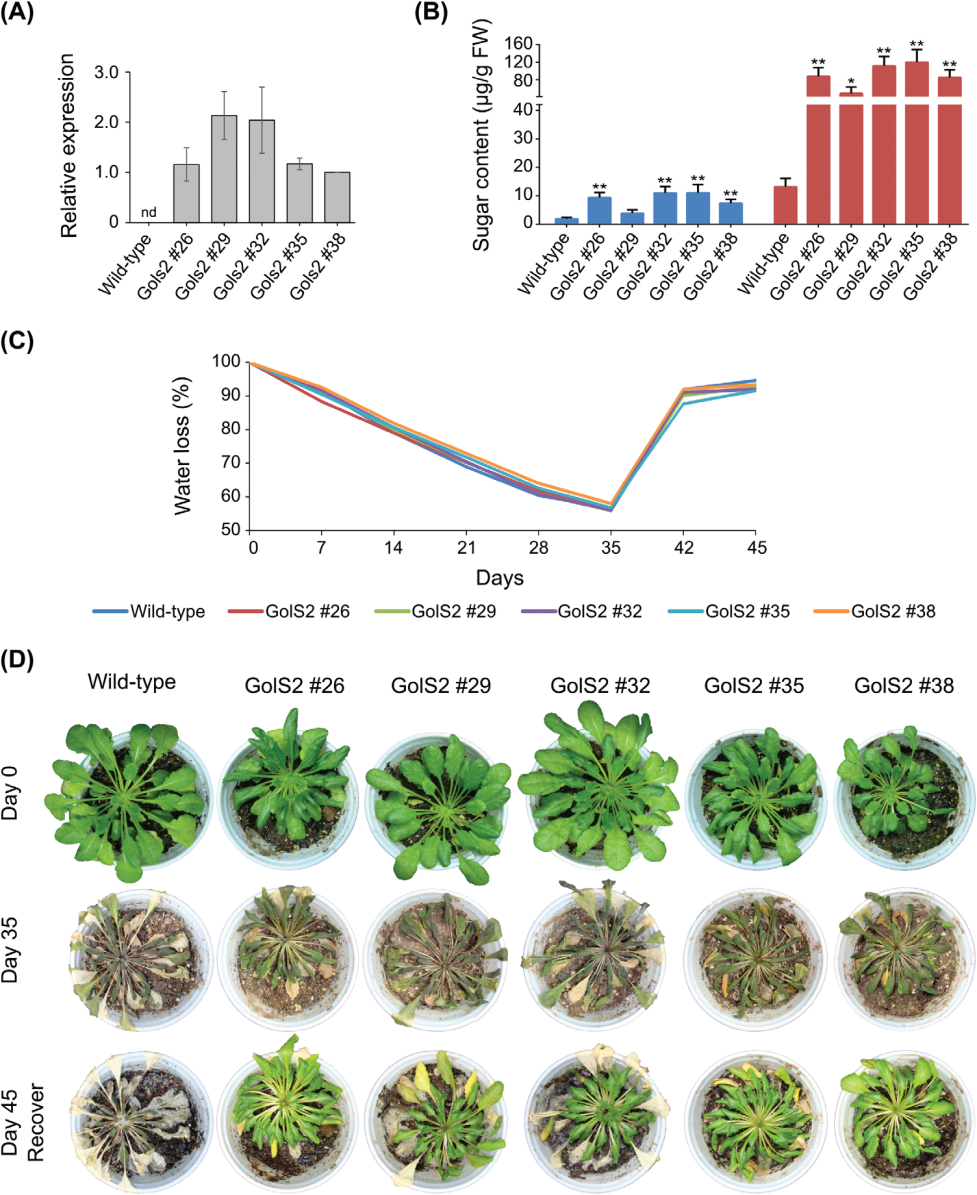
Evaluation of Arabidopsis plants expressing *MdGolS2*. (A) Real-time PCR analysis of the transgene transcript levels in Arabidopsis leaves. Gene expression was plotted relatively to GolS2 no. 38 transcript levels. Standard error bars are shown. nd, not-detected. (B) Galactinol (blue) and raffinose (red) levels in Arabidopsis leaves. Asterisks indicate statistical significance between the transgenic line and the wild-type plant (*t* test: *P<0.1, **P<0.05). (C) Water loss in plant pots after water deprivation. (D) Plant phenotype during the water deficit assay. Two-month-old plants were deprived of water for 35 days. Thereafter, they were rehydrated for ten days prior to evaluation. (This figure is available in color at *JXB* online.)

A preliminary water deficit assay was performed in wild-type plants to identify the number of days needed to cause lethal damage. After 24–27 days without water, wild-type plants were unable to survive even after rehydration. Five independent transgenic lines were recovered and analysed (Fig. 5A). Galactinol and raffinose contents were measured before treatment, and wild-type plants showed lower amounts of galactinol and raffinose in comparison with transgenic plants (Fig. 5B). Two-month-old transformed and wild-type plants had their water supply removed for 35 days to perform the water deficit assay. Water loss was independently measured in each pot once a week. Around 10% of water content was lost every week during the treatment, with starting values recovered after rehydration (Fig. 5C). Only the transgenic lines survived after rehydration (Fig. 5D). All transgenic lines followed their normal growth cycle after treatment, producing viable flowers, siliques, and seeds.

## Discussion

Galactinol synthase is a key enzyme in the biosynthetic pathway of RFOs, which are carbohydrates that play important roles in carbon storage, signal transduction, and osmoprotection, among others (Elsayed *et al.*, 2014; Sengupta *et al.*, 2015). Although several studies identified *GolS* genes and RFO levels as up-regulated during bud dormancy, suggesting that the seasonal mobilization of these carbohydrates could lead to bud integrity protection (Cox and Stushnoff, 2001; Derory *et al.*, 2006; Santamaría *et al.*, 2011; Liu *et al.*, 2012; Unda *et al.*, 2012; Ibáñez *et al.*, 2013; Falavigna *et al.*, 2014; Yue *et al.*, 2015; Andersen *et al.*, 2017), their direct relationship had still not been assessed. Within this context, evolutionary analyses of *GolS* genes in the Rosaceae family were performed followed by the characterization of *MdGolS* genes during bud dormancy, including the functional characterization of *MdGolS2*, a strong candidate to regulate the seasonal accumulation of galactinol and RFOs during growth arrest in apple.

The number of predicted gene models coding for GolS in apple (eight) is similar to that found in Arabidopsis (eight) and poplar (nine) genomes (Yin *et al.*, 2010). All MdGolS proteins showed a putative serine phosphorylation site around amino acid position 265, which is a characteristic feature of nearly all GolS proteins reported so far (Taji *et al.*, 2002). The prevailing exon/intron structure of *MdGolS* genes is the same as that observed for Arabidopsis and poplar *GolS* sequences (Fig. 1). Interestingly, only five *MdGolS* genes were expressed under the conditions analysed (see Supplementary Fig. S1), and in agreement with this organ-selective *GolS* expression observed in apple, Arabidopsis *AtGolS5*, *AtGolS6*, *AtGolS7*, *AtGolS9*, and *AtGolS10* are almost undetectable in some organs such as leaves (Nishizawa *et al.*, 2008). A wide range of number of *GolS* genes was identified in the Rosaceae family, with apple and European pear showing the highest numbers. One possible explanation is that apples and pears experienced a recent WGD event that is not shared by other Rosaceous genera such as *Prunus* and *Fragaria* (Jung *et al.*, 2012; Zhang *et al.*, 2012). Additionally, besides the apple, Arabidopsis, and poplar *GolS* gene structural similarities, all these three species were subjected to recent WGDs (Velasco *et al.*, 2010; Jiao *et al.*, 2011; Daccord *et al.*, 2017), and this may be indicative that this kind of duplication event was a driving force in the evolution of this particular class of genes.

To gain insights into such assumptions, evolutionary analyses were performed across Rosaceous species. A good robustness was obtained between analyses, due to several orthologs being identified in both the phylogenetic and collinear approaches (Table 3; Fig. 2; Supplementary Fig. S3). Moreover, several collinear regions containing *GolS* genes provided good examples of syntenic relationships among Rosaceae genomes (Jung *et al.*, 2012; Zhang *et al.*, 2012; Wu *et al.*, 2013). Interestingly, all *GolS* paralogs from Rosaceous genera had their duplicated gene origin annotated as WGDs. The *K*_s_-dating estimation of these paralogs allowed us to identify two distinct group values that can be related to different duplication events (Table 2). The first one was composed of some apple and pear paralogs and displayed *K*_s_ values lower than 0.25, which corresponds to a recent WGD that is only shared by these two species in the Rosaceae family (Velasco *et al.*, 2007; Xiang *et al.*, 2017). The second group showed *K*_s_ values around 1.5 and was identified among *GolS* paralogs from *Malus*, *Prunus*, and *Fragaria* genomes. Similar *K*_s_ values were also obtained for *GolS* paralogs of grapevine (Supplementary Table S4), a species that has only undergone the triplication event without subsequent polyploidy events (Tang *et al.*, 2008). In addition, it is estimated that *K*_s_ values around 1.5 originated after the divergence of *Amborella* and the rest of the angiosperms (Jiao *et al.*, 2011). In this context, *GolS* paralogs from apple, Japanese apricot, peach, woodland strawberry, and grapevine with *K*_s_ values around 1.5 likely originated in the triplication event. Specifically for apple, the expansion of *GolS* genes was driven by both triplication and recent WGD events, with no evidences of tandem rearrangements such as the ones observed in grapevine.

In general, paleopolyploidy events may provide opportunities for positive selection, generating copies of genes that persist in the course of evolution by retaining advantageous mutations (Yang and Bielawski, 2000; Zhang, 2003). The *K*_a_/*K*_s_ ratios of the duplicated *GolS* genes in the Rosaceae family indicated a strong purifying selection after WGDs (Table 2). In several plants, genes derived from older duplication events also tended to have experienced stronger purifying selection (Zou *et al.*, 2009; Du *et al.*, 2013; Körbes *et al.*, 2016). The maintenance of several GolS isoforms, together with the slow mutation rate in duplicated genes, may be explained by the importance of the roles of galactinol and RFOs in different cellular functions. Within this context, one possible explanation is that the differential *GolS* gene expression would be more important to define gene function than the protein structure itself, and this is a common evolutionary fate of duplicate genes called subfunctionalization (Zhang, 2003). This is a potentially important trend during evolution, where two genes with identical functions are unlikely to be stably maintained in the genome unless there is a division of gene expression after duplication (Zhang, 2003).

In fact, distinct expression patterns were observed for *MdGolS* genes in samples from different developmental stages (Fig. 3), as well as during bud dormancy progression (Fig. 4). *MdGolS1*, *3*, and *4* presented highest transcript levels in mature seeds, agreeing with previous reports that identified *GolS* accumulation during seed development (Elsayed *et al.*, 2014; Sengupta *et al.*, 2015; Salvi *et al.*, 2016). The proposed role of GolS would be to increase seed RFO amounts, offering desiccation tolerance and energy during germination (Blöchl *et al.*, 2007; Elsayed *et al.*, 2014; Sengupta *et al.*, 2015). Recently, supplementation with galactose, a carbohydrate that integrates the RFO pathway, was shown to partially rescue a delayed seed germination phenotype observed in double knockout plants for raffinose synthase 4 and 5 (Gangl and Tenhaken, 2016). It has been proposed that galactose may repress the expression of *AtPIF6* (phytochrome interacting factor 6), an important regulator in establishing the level of primary seed dormancy, and that RFOs would be a storage complex of galactose (Gangl and Tenhaken, 2016). Interestingly, almost all *MdGolS* genes were mainly expressed in dormant seeds or buds (Fig. 3), and common pathways between bud and seed dormancy were already reported (Leida *et al.*, 2012; Wang *et al.*, 2016*a*). Our findings reinforce that bud and seed dormancy may share similar physiological mechanisms, with *GolS* genes playing roles in both processes.

This finding prompted us to characterize the *MdGolS* gene expression dynamics, as well as the galactinol and RFO contents, in dormant buds during an annual growth and dormancy cycle (Fig. 4). *MdGolS1* and *MdGolS2* reached highest steady-state mRNA levels during winter. In agreement, galactinol and raffinose levels showed the same seasonal pattern and a high positive correlation with *MdGolS1* and *MdGolS2* transcript levels (Table 4). Similar *GolS* transcript patterns during dormancy were identified in blackcurrant, chestnut, hybrid poplar, pear, and sessile oak (Derory *et al.*, 2006; Santamaría *et al.*, 2011; Liu *et al.*, 2012; Unda *et al.*, 2012; Ibáñez *et al.*, 2013; Andersen *et al.*, 2017), whereas a similar raffinose content pattern was identified in blackcurrant and trembling aspen (Cox and Stushnoff, 2001; Andersen *et al.*, 2017). The probable function of galactinol and RFOs during bud dormancy may be related to their osmolyte properties, mainly during dehydration (Elsayed *et al.*, 2014; Sengupta *et al.*, 2015). Indeed, dormant buds have to cope with a limited free water content during dormancy, employing several strategies to diminish damage due to this condition (de Faÿ *et al.*, 2000; Améglio *et al.*, 2002; Kalcsits *et al.*, 2009).

The evolutionary and transcriptional data gathered for *MdGolS* genes suggest that rather than playing different functions, these genes may exert similar functions but in different tissues and organs. While *MdGolS2* was 627-fold more expressed in dormant buds in comparison with apple seeds, *MdGolS1*, *MdGolS3*, and *MdGolS4* were mainly expressed in apple seeds (Fig. 3). This suggests that *MdGolS1*, *MdGolS3*, and *MdGolS4* may have retained the already described role of *GolS* genes in seed development (Blöchl *et al.*, 2007; Elsayed *et al.*, 2014; Sengupta *et al.*, 2015), while *MdGolS2* might play a role during bud dormancy. The same model of subfunctionalization has already been observed in several duplicated genes from many species (Zhang, 2003; Fawcett *et al.*, 2009). Taken together, our data suggest that the recent origin of *MdGolS2* resulted in a specialized adaptive role during apple bud dormancy (Tables 2 and 4; Fig. 4). In order to evaluate if *MdGolS2*, galactinol, and raffinose integrate one of the mechanisms conferring protection to dormant buds against a limited water supply, this gene was functionally characterized in Arabidopsis plants.

Transgenic Arabidopsis lines expressing *MdGolS2* were generated and submitted to a severe water deficit assay (Fig. 5). Transgenic plants accumulated higher amounts of galactinol and RFOs in comparison with wild-type plants, mimicking what is observed in apple buds during dormancy. After the water withdrawal assay, only the transgenic plants were capable of surviving. This provides evidence that one of MdGolS2’s functions during bud dormancy is to synthesize galactinol, leading to increased amounts of RFOs, which will act together in bud protection against limited water supply. Transgenic expression of *GolS* genes from several different species was also capable of conferring tolerance to water deficit, freezing, heavy metal, or salt stress (Taji *et al.*, 2002; Nishizawa *et al.*, 2008; Wang *et al.*, 2009; Song *et al.*, 2013; Sun *et al.*, 2013; Honna *et al.*, 2016; Wang 2016*b*; Yu *et al.*, 2017; Selvaraj *et al.*, 2017). This increased stress tolerance may be due to the ability of galactinol and RFOs to scavenge hydroxyl radicals as well as their osmolyte properties, maintaining cell turgor, and stabilizing cell proteins and membranes (Nishizawa *et al.*, 2008; Elsayed *et al.*, 2014; Salvi *et al.*, 2016).

The roles of starch, soluble sugars and sugar alcohols during dormancy have already been addressed in several reports, suggesting that carbohydrate metabolism is a key feature during dormancy, especially dormancy release (Maurel *et al.*, 2004; Anderson *et al.*, 2005; Marafon *et al.*, 2011; Ito *et al.*, 2013; Zhuang *et al.*, 2015; Hussain *et al.*, 2016; Andersen *et al.*, 2017). Our findings add galactinol and RFOs to the list of sugars with important roles during bud dormancy. In apple, galactinol and raffinose integrate a series of adaptive mechanisms that act together during dormancy to protect buds from the intrinsic limited water supply that occurs during winter. MdGolS2 is likely the main enzyme responsible for their seasonal accumulation, although we cannot exclude that other MdGolS, especially MdGolS1, may also contribute for RFO synthesis during bud dormancy. Moreover, the reduced levels of these carbohydrates prior to budbreak suggest that they are being used as an energy source, in a similar trend to previous observations with other sugars (Maurel *et al.*, 2004; Zhuang *et al.*, 2015; Hussain *et al.*, 2016). The accumulation of metabolites that can be used as protectors as well as an energy source is a highly advantageous evolutionary feature. WGD events have driven the evolution and diversification of *GolS* genes in angiosperms, especially in the Rosaceae family, resulting in the generation and further maintenance of several copies of these genes. It is tempting to speculate that the appearance of new structures and developmental programs, such as buds and dormancy, required the adaptation of already established molecular pathways. Possibly, the differential gene expression patterns of *GolS* genes improved plant adaptation to unsuitable environments, prompting the development of complex angiosperms.

## Supplementary Data

Supplementary data are available at *JXB* online.

Fig. S1. Agarose gel electrophoretic result of PCR analysis of *MdGolS* genes in ‘Gala Baigent’ apple trees.

Fig. S2. Multiple alignments of *GolS* CDS predicted in the apple genome with those cDNA sequences amplified by RACE.

Fig. S3. Collinearity analyses between apple and other Rosaceous species.

Table S1. Sampling dates, corresponding season and chilling hours accumulated by ‘Fuji Standard’ buds.

Table S2. List of primers employed in this work.

Table S3. Sequences used for synteny and phylogenetic analyses.

Table S4. *K*_s_-dating of grapevine *GolS* paralogs.

Supplementary Figures S1-S3

Supplementary Tables S1-S4

## Abbreviations:

CBF: C-repeat binding transcription factors
GolS: galactinol synthase
*K*_a_: non-synonymous substitution rate
*K*_s_: synonymous substitution rates
MRM: multiple reaction monitoring
MYA: million years ago
RFO: raffinose family oligosaccharide
WGD: whole-genome duplication.
