# Supplementary material for: Evolutionary diversification of galactinol synthases in Rosaceae: adaptive roles of galactinol and raffinose during apple bud dormancy
Source: J Exp Bot. 2018 Jan 24;69(5):1247–59. doi: 10.1093/jxb/erx451 (PMC6018919; doi:10.1093/jxb/erx451)

Evolutionary diversification of Rosaceous galactinol synthases: adaptive roles of galactinol and raffinose during bud dormancy in apple

Vítor da Silveira Falavigna, Diogo Denardi Porto, Yohanna Evelyn Miotto, Henrique Pessoa dos Santos, Paulo Ricardo Dias de Oliveira, Márcia Margis-Pinheiro, Giancarlo Pasquali, Luís Fernando Revers

Supplementary figures

Fig. S1. Agarose gel electrophoretic result PCR analysis of *MdGolS* genes in 'Gala Baigent' apple trees. Genomic DNA (lane 1), cDNA pool (lane 2) or water (lane 3), were submitted to RT-PCR. M, GeneRuler® 1 kb Plus DNA Ladder (Thermo Fisher Scientific, Waltham, USA). Arrows indicate amplification products from cDNA samples. The *MDH* gene was used as positive control. The expected amplicon size for each gene is presented in Table S2.

Fig. S2. Multiple alignments of *GolS* CDS predicted in the apple genome with those cDNA sequences amplified by RACE.

Fig. S3. Collinearity analyses between apple and other Rosaceous species. Only chromosomes or scaffolds, when appropriate, containing *GolS* genes are shown. Collinear blocks with *GolS* genes are shown as black lines, whereas the remaining collinear blocks are shown in gray lines. Analysis performed included chromosomes/scaffolds from apple and **(A)** Chinese pear; **(B)** European pear; **(C)** Japanese apricot; **(D)** peach; and **(E)** woodland strawberry.

Supplementary Figure S1

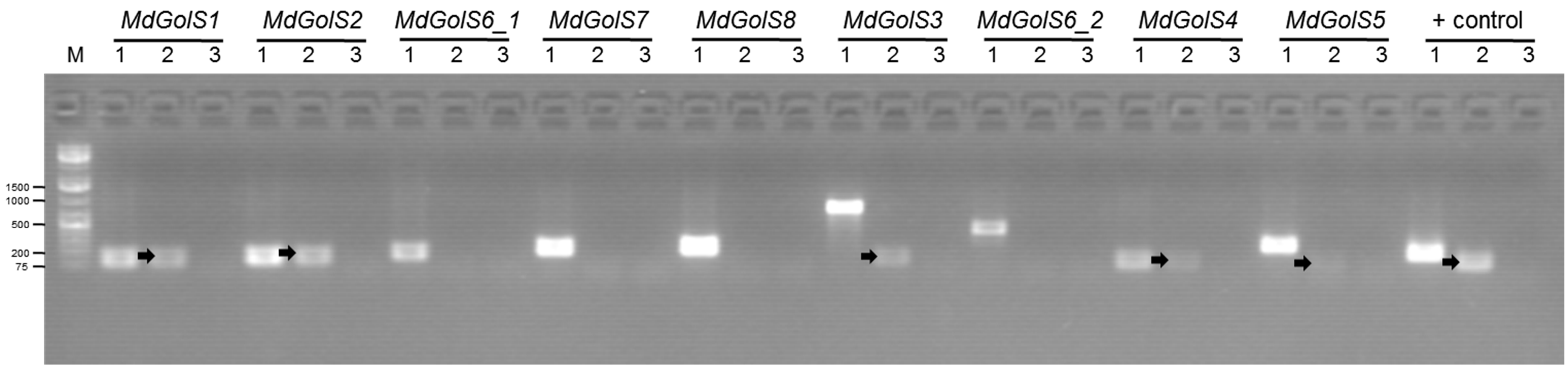

Supplementary Figure S2

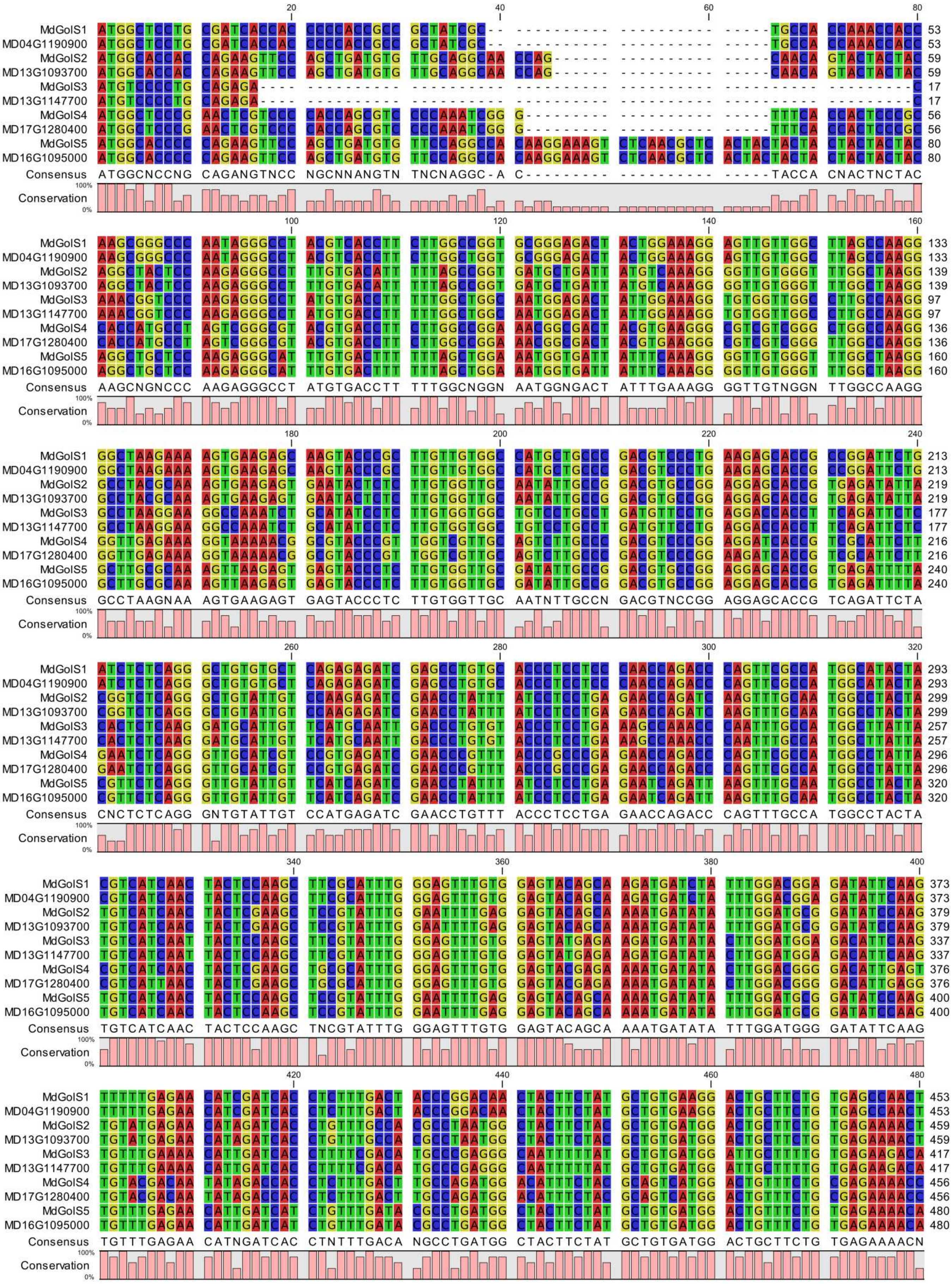

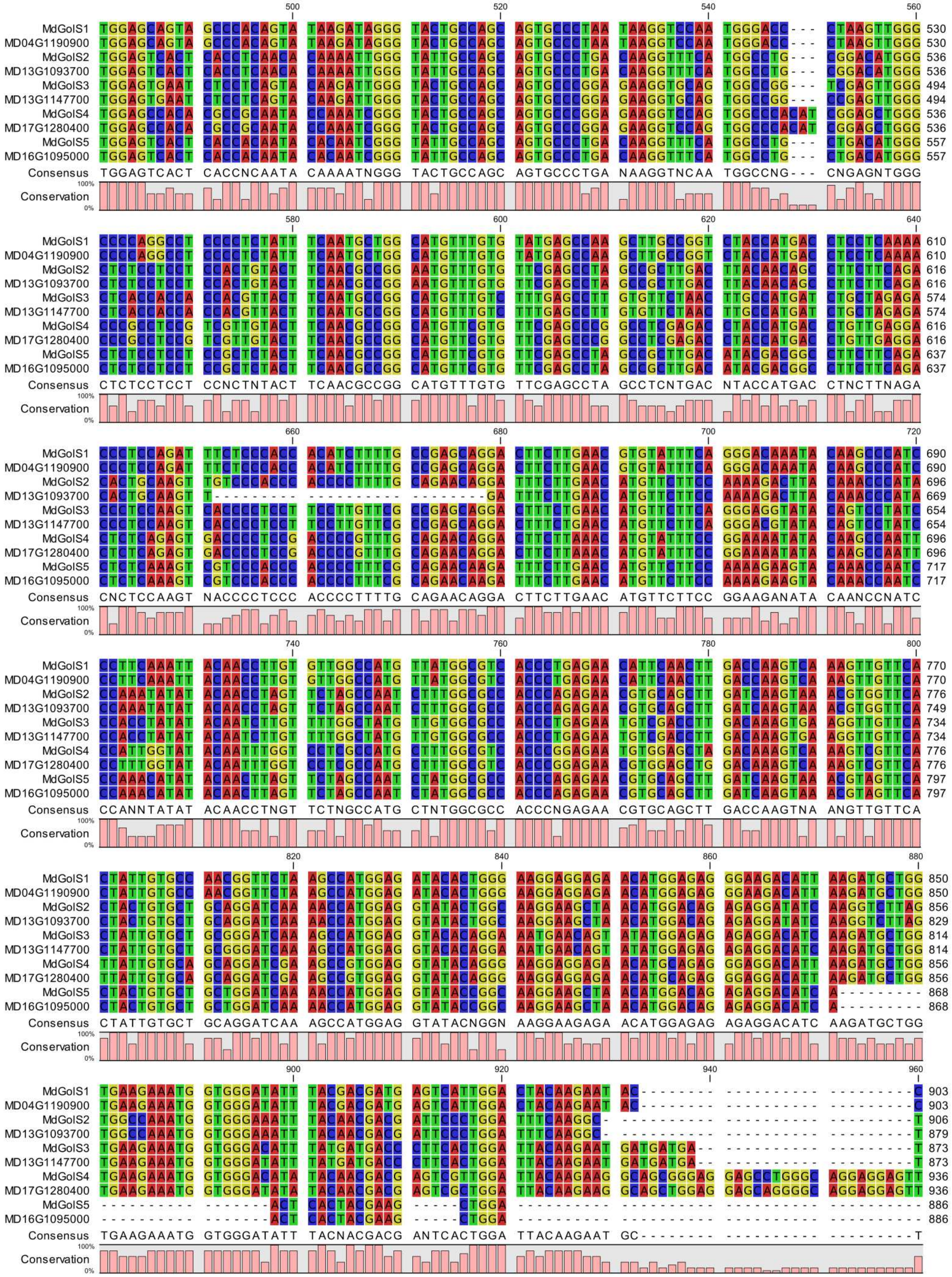

500 520 540 560
MdGolS1 TGGAGCAGTA GCCCACAGTA TAAGATAGGG TACTGCCAGC AGTGCCCTAA TAAGGTCCAA TGGGACC--- CTAAGTTGGG 530
MD04G1190900 TGGAGCAGTA GCCCACAGTA TAAGATAGGG TACTGCCAGC AGTGCCCTAA TAAGGTCCAA TGGGACC--- CTAAGTTGGG 530
MdGolS2 TGGAGTCACT CACCTCAACA CAAAATTGGG TATTGCCAGC AGTGCCCTGA CAAGGTTTCA TGGCCTG--- CGGACATGGG 536
MD13G1093700 TGGAGTCACT CACCTCAACA CAAAATTGGG TATTGCCAGC AGTGCCCTGA CAAGGTTTCA TGGCCTG--- CGGACATGGG 536
MdGolS3 TGGAGTGAAT CTCCTCAGTA CAAGATTGGG TACTGCCAGC AGTGCCCGGA GAAGGTGCAG TGGCCGG--- TCGAGTTGGG 494
MD13G1147700 TGGAGTGAAT CTCCTCAGTA CAAGATTGGG TACTGCCAGC AGTGCCCGGA GAAGGTGCAG TGGCCGG--- CCGAGTTGGG 494
MdGolS4 TGGAGCCACA CGCCGCAATA CCAAATCGGG TACTGCCAGC AGTGCCCGGA GAAGGTCCAG TGGCCCACAT CGGAGCTGGG 536
MD17G1280400 TGGAGCCACA CGCCGCAATA CCAAATCGGG TACTGCCAGC AGTGCCCGGA GAAGGTCCAG TGGCCCACAT CGGAGCTGGG 536
MdGolS5 TGGAGTCACT CACCACAATA CACAATCGGG TATTGCCAGC AGTGCCCTGA CAAGGTTTCA TGGCCTG--- CTGACATGGG 557
MD16G1095000 TGGAGTCACT CACCACAATA CACAATCGGG TATTGCCAGC AGTGCCCTGA CAAGGTTTCA TGGCCTG--- CTGACATGGG 557
Consensus TGGAGTCACT CACCNCAATA CAAAATNGGG TACTGCCAGC AGTGCCCTGA NAAGGTNCAA TGGCCNG--- CNGAGNTGGG
Conservation 100% 0%
580 600 620 640
MdGolS1 CCCCAGGCCT CCCCTCTATT TCAATGCTGG CATGTTTGTG TATGAGCCAA GCTTGCCGGT CTACCATGAC CTCCTCAAAA 610
MD04G1190900 CCCCAGGCCT CCCCTCTATT TCAATGCTGG CATGTTTGTG TATGAGCCAA GCTTGCCGGT CTACCATGAC CTCCTCAAAA 610
MdGolS2 CTCTCCTCCT CCACTGTACT TCAACGCCGG AATGTTTGTG TTCGAGCCTA GCCGCTTGAC TTACAACAGC CTTCTTCAGA 616
MD13G1093700 CTCTCCTCCT CCACTGTACT TCAACGCCGG AATGTTTGTG TTCGAGCCTA GCCGCTTGAC TTACAACAGC CTTCTTCAGA 616
MdGolS3 CTCACCACCA CCACGTTACT TCAATGCCGG CATGTTTGTC TTTGAGCCTT GTGTTCTAAC TTGCCATGAT CTGCTAGAGA 574
MD13G1147700 CTCACCACCA CCACGTTACT TCAATGCCGG CATGTTTGTC TTTGAGCCTT GTGTTCTAAC TTGCCATGAT CTGCTAGAGA 574
MdGolS4 CCCGCCTCCG TCGTTGTACT TCAACGCCGG CATGTTCGTG TTCGAGCCCG GCCTCGAGAC CTACCATGAC CTGTTGAGGA 616
MD17G1280400 CCCGCCTCCG TCGTTGTACT TCAACGCCGG CATGTTCGTG TTCGAGCCCG GCCTCGAGAC CTACCATGAC CTGTTGAGGA 616
MdGolS5 CTCTCCTCCT CCGCTCTACT TCAACGCCGG CATGTTCGTG TTCGAGCCTA GCCGCTTGAC ATACGACGGC CTTCTTCAGA 637
MD16G1095000 CTCTCCTCCT CCGCTCTACT TCAACGCCGG CATGTTCGTG TTCGAGCCTA GCCGCTTGAC ATACGACGGC CTTCTTCAGA 637
Consensus CTCTCCTCCT CCNCTNTACT TCAACGCCGG CATGTTTGTG TTCGAGCCTA GCCTCNTGAC NTACCATGAC CTNCTTNAGA
Conservation 100% 0%
660 680 700 720
MdGolS1 CCCTCCAGAT TTCTCCCACC ACATCTTTTG CCGAGCAGGA CTTCTTGAAC GTGTATTTCA GGGACAAATA CAAGCCCATC 690
MD04G1190900 CCCTCCAGAT TTCTCCCACC ACATCTTTTG CCGAGCAGGA CTTCTTGAAC GTGTATTTCA GGGACAAATA CAAGCCCATC 690
MdGolS2 CACTGCAAGT TGTCCCACCC ACCCCTTTTG CAGAACAGGA TTTCTTGAAC ATGTTCTTCC AAAAGACTTA CAAACCCATA 696
MD13G1093700 CACTGCAAGT T--------- ---------- --------GA TTTCTTGAAC ATGTTCTTCC AAAAGACTTA CAAACCCATA 669
MdGolS3 CCCTCCAAGT CACCCCTCCT TCCTTGTTCG CCGAGCAGGA CTTTCTGAAC ATGTTCTTCA GGGAGGTATA CAGTCCTATC 654
MD13G1147700 CCCTCCAAGT CACCCCTCCT TCCTTGTTCG CCGAGCAGGA CTTTCTGAAC ATGTTCTTCA GGGACGTATA CAGTCCTATC 654
MdGolS4 CTCTCAGAGT GACCCCTCCG ACCCCGTTTG CAGAACAGGA CTTCTTAAAC ATGTATTTCC GGAAAATATA CAAGCCAATT 696
MD17G1280400 CTCTCAGAGT GACCCCTCCG ACCCCGTTTG CAGAACAGGA CTTCTTAAAC ATGTATTTCC GGAAAATATA CAAGCCAATT 696
MdGolS5 CTCTCAAAGT CGTCCCACCC ACCCCTTTCG CAGAACAAGA TTTCTTGAAC ATGTTCTTCC AAAAGAAGTA CAAACCAATC 717
MD16G1095000 CTCTCAAAGT CGTCCCACCC ACCCCTTTCG CAGAACAAGA TTTCTTGAAC ATGTTCTTCC AAAAGAAGTA CAAACCAATC 717
Consensus CNCTCCAAGT NACCCCTCCC ACCCCTTTTG CAGAACAGGA CTTCTTGAAC ATGTTCTTCC GGAAGANATA CAANCCNATC
Conservation 100% 0%
740 760 780 800
MdGolS1 CCTTCAAATT ACAACCTTGT GTTGGCCATG TTATGGCGTC ACCCTGAGAA CATTCAACTT GACCAAGTCA AAGTTGTTCA 770
MD04G1190900 CCTTCAAATT ACAACCTTGT GTTGGCCATG TTATGGCGTC ACCCTGAGAA CATTCAACTT GACCAAGTCA AAGTTGTTCA 770
MdGolS2 CCAAATATAT ACAACCTAGT TCTAGCCAAT CTTTGGCGCC ACCCAGAGAA CGTGCAGCTT GATCAAGTAA ACGTGGTTCA 776
MD13G1093700 CCAAATATAT ACAACCTAGT TCTAGCCAAT CTTTGGCGCC ACCCAGAGAA CGTGCAGCTT GATCAAGTAA ACGTGGTTCA 749
MdGolS3 CCACCTATAT ACAATCTTGT TTTGGCTATG TTGTGGCGCC ACCCTGAGAA TGTCGACCTT GACAAAGTGA AGGTTGTTCA 734
MD13G1147700 CCACCTATAT ACAATCTTGT TTTGGCTATG TTGTGGCGCC ACCCTGAGAA TGTCGACCTT GACAAAGTGA AGGTTGTTCA 734
MdGolS4 CCATTGGTAT ACAATTTGGT CCTCGCCATG CTTTGGCGTC ACCCGGAGAA TGTGGAGCTA GACAAAGTCA AAGTCGTTCA 776
MD17G1280400 CCTTTGGTAT ACAATTTGGT CCTCGCCATG CTTTGGCGTC ACCCGGAGAA CGTGGAGCTG GACAAAGTCA AAGTCGTTCA 776
MdGolS5 CCAAACATAT ACAACTTAGT TCTAGCCAAT CTATGGCGCC ACCCAGAGAA CGTGCAGCTT GATCAAGTAA ACGTAGTTCA 797
MD16G1095000 CCAAACATAT ACAACTTAGT TCTAGCCAAT CTATGGCGCC ACCCAGAGAA CGTGCAGCTT GATCAAGTAA ACGTAGTTCA 797
Consensus CCANNTATAT ACAACCTNGT TCTNGCCATG CTNTGGCGCC ACCCNGAGAA CGTGCAGCTT GACCAAGTNA ANGTTGTTCA
Conservation 100% 0%
820 840 860 880
MdGolS1 CTATTGTGCC AACGGTTCTA AGCCATGGAG ATACACTGGG AAGGAGGAGA ACATGGAGAG GGAAGACATT AAGATGCTGG 850
MD04G1190900 CTATTGTGCC AACGGTTCTA AGCCATGGAG ATACACTGGG AAGGAGGAGA ACATGGAGAG GGAAGACATT AAGATGCTGG 850
MdGolS2 CTACTGTGCT GCAGGATCAA AACCATGGAG GTATACTGGC AAGGAAGCTA ACATGGACAG AGAGGATATC AAGGTCTTAG 856
MD13G1093700 CTACTGTGCT GCAGGATCAA AACCATGGAG GTATACTGGC AAGGAAGCTA ACATGGACAG AGAGGATATC AAGGTCTTAG 829
MdGolS3 CTATTGTGCT GCGGGATCAA AGCCATGGAG GTACACAGGA AATGAACAGT ATATGGAGAG AGAGGACATC AAGATGCTGG 814
MD13G1147700 CTATTGTGCT GCGGGATCAA AGCCATGGAG GTACACAGGA AATGAACAGT ATATGGAGAG AGAGGACATC AAGATGCTGG 814
MdGolS4 TTATTGTGCA GCAGGATCGA AGCCGTGGAG GTATACAGGG AAGGAGGAGA ACATGCAGAG GGAGGACATT AAGATGCTGG 856
MD17G1280400 TTATTGTGCA GCAGGATCGA AGCCGTGGAG GTATACAGGG AAGGAGGAGA ACATGCAGAG GGAGGACATT AAGATGCTGG 856
MdGolS5 CTACTGTGCT GCTGGATCAA AACCATGGAG GTATACCGGC AAGGAAGCTA ACATGGACAG AGAGGACATC A--------- 868
MD16G1095000 CTACTGTGCT GCTGGATCAA AACCATGGAG GTATACCGGC AAGGAAGCTA ACATGGACAG AGAGGACATC A--------- 868
Consensus CTATTGTGCT GCAGGATCAA AGCCATGGAG GTATACNGGN AAGGAAGAGA ACATGGAGAG AGAGGACATC AAGATGCTGG
Conservation 100% 0%
900 920 940 960
MdGolS1 TGAAGAAATG GTGGGATATT TACGACGATG AGTCATTGGA CTACAAGAAT AC-------- ---------- ---------C 903
MD04G1190900 TGAAGAAATG GTGGGATATT TACGACGATG AGTCATTGGA CTACAAGAAT AC-------- ---------- ---------C 903
MdGolS2 TGGCCAAATG GTGGGAAATT TACAACGACG ATTCCCTGGA TTTCAAGGC- ---------- ---------- ---------T 906
MD13G1093700 TGGCCAAATG GTGGGAAATT TACAACGACG ATTCCCTGGA TTTCAAGGC- ---------- ---------- ---------T 879
MdGolS3 TGAAGAAATG GTGGGACATT TATGATGACC CTTCACTGGA TTACAAGAAT GATGATGA-- ---------- ---------T 873
MD13G1147700 TGAAGAAATG GTGGGATATT TATGATGACC CTTCACTGGA TTACAAGAAT GATGATGA-- ---------- ---------T 873
MdGolS4 TGAAGAAATG GTGGGACATA TACAACGACG AGTCGTTGGA TTACAAGAAG GCAGCGGGAG GAGCCTGGGC AGGAGGAGTT 936
MD17G1280400 TGAAGAAATG GTGGGATATA TACAACGACG AGTCGCTGGA TTACAAGAAG GCAGCTGGAG GAGCAGGGGC AGGAGGAGTT 936
MdGolS5 ---------- -------ACT CACTACGAAG -----CTGGA ---------- ---------- ---------- ---------- 886
MD16G1095000 ---------- -------ACT CACTACGAAG -----CTGGA ---------- ---------- ---------- ---------- 886
Consensus TGAAGAAATG GTGGGATATT TACNACGACG ANTCACTGGA TTACAAGAAT GC-------- ---------- ---------T
Conservation 100% 0%

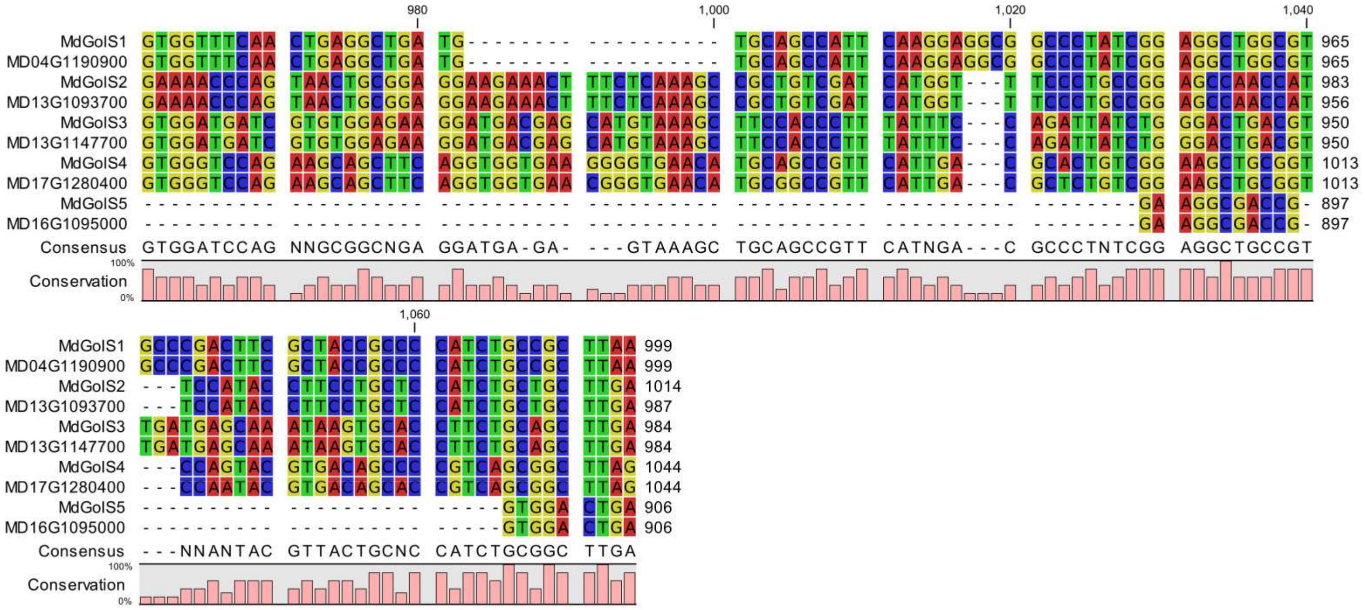
980
1,000
1,020
1,040
MdGolS1 GTGGTTTCAA CTGAGGCTGA TG-------- ---------- TGCAGCCATT CAAGGAGGCG GCCCTATCGG AGGCTGGCGT 965
MD04G1190900 GTGGTTTCAA CTGAGGCTGA TG-------- ---------- TGCAGCCATT CAAGGAGGCG GCCCTATCGG AGGCTGGCGT 965
MdGolS2 GAAAACCCAG TAACTGCGGA GGAAGAAACT TTCTCAAAGC CGCTGTCGAT CATGGT---T TCCCTGCCGG AGCCAACCAT 983
MD13G1093700 GAAAACCCAG TAACTGCGGA GGAAGAAACT TTCTCAAAGC CGCTGTCGAT CATGGT---T TCCCTGCCGG AGCCAACCAT 956
MdGolS3 GTGGATGATC GTGTGGAGAA GGATGACGAG CATGTAAAGC TTCCACCCTT TATTTC---C AGATTATCTG GGACTGACGT 950
MD13G1147700 GTGGATGATC GTGTGGAGAA GGATGACGAG CATGTAAAGC TTCCACCCTT TATTTC---C AGATTATCTG GGACTGACGT 950
MdGolS4 GTGGGTCCAG AAGCAGCTTC AGGTGGTGAA GGGGTGAACA TGCAGCCGTT CATTGA---C GCACTGTCGG AAGCTGCGGT 1013
MD17G1280400 GTGGGTCCAG AAGCAGCTTC AGGTGGTGAA CGGGTGAACA TGCGGCCGTT CATTGA---C GCTCTGTCGG AAGCTGCGGT 1013
MdGolS5 ---------- ---------- ---------- ---------- ---------- ---------- --------GA AGGCGACCG- 897
MD16G1095000 ---------- ---------- ---------- ---------- ---------- ---------- --------GA AGGCGACCG- 897
Consensus GTGGATCCAG NNGCGGCNGA GGATGA-GA- ---GTAAAGC TGCAGCCGTT CATNGA---C GCCCTNTCGG AGGCTGCCGT
100%
Conservation
0%
1,060
MdGolS1 GCCCGACTTC GCTACCGCCC CATCTGCCGC TTAA 999
MD04G1190900 GCCCGACTTC GCTACCGCCC CATCTGCCGC TTAA 999
MdGolS2 ---TCCATAC CTTCCTGCTC CATCTGCTGC TTGA 1014
MD13G1093700 ---TCCATAC CTTCCTGCTC CATCTGCTGC TTGA 987
MdGolS3 TGATGAGCAA ATAAGTGCAC CTTCTGCAGC TTGA 984
MD13G1147700 TGATGAGCAA ATAAGTGCAC CTTCTGCAGC TTGA 984
MdGolS4 ---CCAGTAC GTGACAGCCC CGTCAGCGGC TTAG 1044
MD17G1280400 ---CCAATAC GTGACAGCAC CGTCAGCGGC TTAG 1044
MdGolS5 ---------- ---------- -----GTGGA CTGA 906
MD16G1095000 ---------- ---------- -----GTGGA CTGA 906
Consensus ---NNANTAC GTTACTGCNC CATCTGCGGC TTGA
100%
Conservation
0%

Supplementary Figure S3

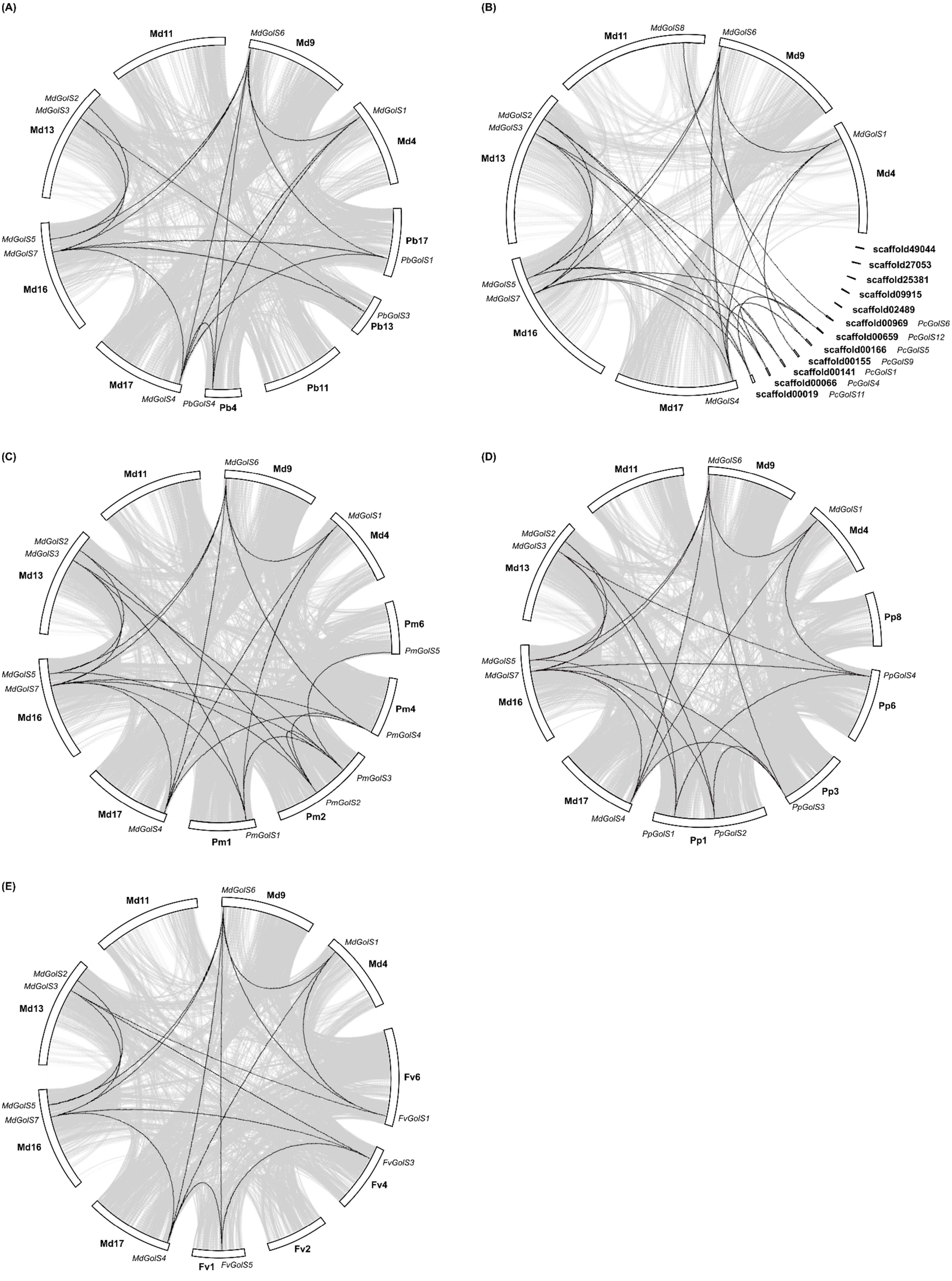

Supplement: Supplementary Figures S1-S3 [file erx451_suppl_supplementary_figures_s1-s3.pdf]
