# Supplementary material for: Evolutionary diversification of galactinol synthases in Rosaceae: adaptive roles of galactinol and raffinose during apple bud dormancy
Source: J Exp Bot. 2018 Jan 24;69(5):1247–59. doi: 10.1093/jxb/erx451 (PMC6018919; doi:10.1093/jxb/erx451)
Supplement: Supplementary Tables S1-S4 [file erx451_suppl_supplementary_tables_s1-s4.pdf]

Evolutionary diversification of Rosaceous galactinol synthases: adaptive roles of galactinol and raffinose during bud dormancy in apple

Vítor da Silveira Falavigna, Diogo Denardi Porto, Yohanna Evelyn Miotto, Henrique Pessoa dos Santos, Paulo Ricardo Dias de Oliveira, Márcia Margis-Pinheiro, Giancarlo Pasquali, Luís Fernando Revers

Supplementary tables

Table S1. Sampling dates, corresponding season and chilling hours (CH, number of hours below 7.2ºC) accumulated by 'Fuji Standard' buds.

Date	Corresponding season	CH
01/21/2009	Summer	0
03/26/2009	Autumn	0
05/27/2009	Autumn	76
06/30/2009	Winter	278
07/30/2009	Winter	450
09/09/2009	Winter	528
11/25/2009	Spring	0
02/01/2010	Summer	0

Table S2. List of primers employed in this work.

Name	Primers	Amplicon size on cDNA samples (bp)	Amplicon size on gDNA samples (bp)
MdGolS1	F: CGATCACCTCTTTGACTACCC*<br>R: TACTGTGGGCTACTGCTCCA*	87	87
MdGolS2	F: CAATACAGCCCTGAGACCG*<br>R: GCCTACGCAAAGTGAAGAGTG*	99	99
	CGAGCCTAGCCGCTTGAC*	-	-
MdGolS3	F: GCCACCCTGAGAATGTCG*<br>R: TCTTGATGTCCTCTCTCTCCA*	117	488
	CGCCTCCGTCGTTGTACTTC*	-	-
MdGolS4	F: CCAAGGGGTTGAGAAAGGTA*<br>R: CCTGAGATTCAAGAATGCGA*	96	96
	CGAGGGCAATTTTTATGCTGT*	-	-
MdGolS5	F: GGATTGGTTTGTACTTCTTTTGGA*<br>R: AGCCTAGCCGCTTGACATAC*	117	269
MdGolS6_1	F: TCTCGGGTGGGTAAACGG<br>R: CAAGGGATTGAGGAAGGTCA	137	137
MdGolS6_2	F: GCTTCGCCACTGCTTACG<br>R: ACCTGAATGTCCCGTCCA	71	457
MdGolS7	F: GAGAAGACATGGAGTGCGTCTC<br>R: CGAGGAAGGAGGGGTAACTTC	156	185
MdGolS8	F: CGTTTCCGTCCAAGAAGATC<br>R: GCACAGTGAAAGAGATTGAGCC	114	212
MdGolS2_CDS	F:CACCCTCGAGATGGCACCACCAGAAGTTCC<br>R:AGATCTTCAAGCAGCAGATGGAGCAG	1014	-
AtAct2	F: GACCTTGCTGGACGTGACCTTAC<br>R: GTAGTCAACAGCAACAAAGGAGAGC	135	135
AtCOP1	F: GCTCGGCATGTGTCAAAA<br>R: CCACTCAGCGCATCCTTC	500	895

Primers marked with an asterisk were also employed in amplification and sequencing of RACE products.

Table S3. Sequences used for synteny and phylogenetic analyses.

Name	Accession number	Plant	Database
FaGolS1	FANhyb_icon00000194_a.1.g00001.1/partial	Strawberry	*F ananassa* FANhybrid r1.2[a]
FaGolS2	FANhyb_icon00033720_a.1.g00001.1/partial	Strawberry	*F ananassa* FANhybrid r1.2[a]
FaGolS3	FANhyb_rscf00000009.1.g00008.1	Strawberry	*F ananassa* FANhybrid r1.2[a]
FaGolS4	FANhyb_rscf00000149.1.g00001.1	Strawberry	*F ananassa* FANhybrid r1.2[a]
FaGolS5	FANhyb_rscf00000335.1.g00001.1	Strawberry	*F ananassa* FANhybrid r1.2[a]
FaGolS6	FANhyb_rscf00000335.1.g00002.1	Strawberry	*F ananassa* FANhybrid r1.2[a]
FaGolS7	FANhyb_rscf00003859.1.g00001.1	Strawberry	*F ananassa* FANhybrid r1.2[a]
FvGolS1	mrna00243.1-v1.0-hybrid	Woodland strawberry	*Fragaria vesca* v1.0 hybrid[b]
FvGolS2	mrna00244.1-v1.0-hybrid	Woodland strawberry	*Fragaria vesca* v1.0 hybrid[b]
FvGolS3	mrna05015.1-v1.0-hybrid	Woodland strawberry	*Fragaria vesca* v1.0 hybrid[b]
FvGolS4	mrna10568.1-v1.0-hybrid	Woodland strawberry	*Fragaria vesca* v1.0 hybrid[b]
FvGolS5	mrna24544.1-v1.0-hybrid	Woodland strawberry	*Fragaria vesca* v1.0 hybrid[b]
MdGolS1	MD04G1190900	Apple	GDDH13 v1.1[c]
MdGolS2	MD13G1093700	Apple	GDDH13 v1.1[c]
MdGolS3	MD13G1147700	Apple	GDDH13 v1.1[c]
MdGolS4	MD17G1280400	Apple	GDDH13 v1.1[c]
MdGolS5	MD16G1095000	Apple	GDDH13 v1.1[c]
MdGolS6	MD09G1288100	Apple	GDDH13 v1.1[c]
MdGolS7	MD16G1147600	Apple	GDDH13 v1.1[c]
MdGolS8	MD11G1070000	Apple	GDDH13 v1.1[c]
PbGolS1	Pbr008619.1	Chinese white pear	*P. bretschneideri* v1.0[d]
PbGolS2	Pbr011631.1	Chinese white pear	*P. bretschneideri* v1.0[d]
PbGolS3	Pbr014990.1	Chinese white pear	*P. bretschneideri* v1.0[d]
PbGolS4	Pbr028045.1	Chinese white pear	*P. bretschneideri* v1.0[d]
PbGolS5	Pbr033744.1	Chinese white pear	*P. bretschneideri* v1.0[d]
PcGolS1	PCP000396.1	European pear	*Pyrus communis* v1.0[a]
PcGolS2	PCP001555.1	European pear	*Pyrus communis* v1.0[a]
PcGolS3	PCP006472.1	European pear	*Pyrus communis* v1.0[a]
PcGolS4	PCP008315.1	European pear	*Pyrus communis* v1.0[a]
PcGolS5	PCP008443.1	European pear	*Pyrus communis* v1.0[a]
PcGolS6	PCP013831.1	European pear	*Pyrus communis* v1.0[a]
PcGolS7	PCP014143.1	European pear	*Pyrus communis* v1.0[a]
PcGolS8	PCP020723.1	European pear	*Pyrus communis* v1.0[a]
PcGolS9	PCP022796.1	European pear	*Pyrus communis* v1.0[a]
PcGolS10	PCP023911.1	European pear	*Pyrus communis* v1.0[a]
PcGolS11	PCP028844.1	European pear	*Pyrus communis* v1.0[a]
PcGolS12	PCP029528.1	European pear	*Pyrus communis* v1.0[a]
PmGolS1	Pm003196	Japanese apricot	*Prunus mume* v1.0[e]
PmGolS2	Pm006697	Japanese apricot	*Prunus mume* v1.0[e]
PmGolS3	Pm008430	Japanese apricot	*Prunus mume* v1.0[e]
PmGolS4	Pm012900	Japanese apricot	*Prunus mume* v1.0[e]
PmGolS5	Pm020004	Japanese apricot	*Prunus mume* v1.0[e]
PpGolS1	Prupe.1G111300.1.p	Peach	*Prunus persica* v2.1[a]
PpGolS2	Prupe.1G251600.1.p	Peach	*Prunus persica* v2.1[a]
PpGolS3	Prupe.3G005100.1.p	Peach	*Prunus persica* v2.1[a]
PpGolS4	Prupe.6G309400.1.p	Peach	*Prunus persica* v2.1[a]
PpGolS5	Prupe.8G258200.1.p	Peach	*Prunus persica* v2.1[a]
RoGolS1	Bras_G00307	Black raspberry	*R. occidentalis* v1.0.a1[a]
RoGolS2	Bras_G07040	Black raspberry	*R. occidentalis* v1.0.a1[a]
RoGolS3	Bras_G08334	Black raspberry	*R. occidentalis* v1.0.a1[a]
RoGolS4	Bras_G24221	Black raspberry	*R. occidentalis* v1.0.a1[a]
RoGolS5	Bras_G27322	Black raspberry	*R. occidentalis* v1.0.a1[a]

[a]GDR; [b]PHYTOZOME; [c]The Apple Genome and Epigenome; [d]GigaDB; [e]NCBI;

Table S4. Ks-dating of grapevine *GolS* paralogs.

**Duplicated genes**	**Ks-dating of the collinear block**	**Duplicated gene origin**
VIT_01s0127g00470/VIT_07s0005g01970	1.68	WGD
VIT_14s0060g00730/VIT_07s0005g01970	1.11	WGD
VIT_05s0020g00330/VIT_07s0005g01970	0.99	WGD
VIT_05s0077g00430/VIT_07s0005g01970	1.33	WGD
VIT_14s0060g00730/VIT_05s0020g00330	1.49	WGD
VIT_14s0060g00730/VIT_05s0077g00430	1.14	WGD

Sequences were identified in the *Vitis vinifera* 12X Database V1 available at CRIBI (http://genomes.cribi.unipd.it/grape/).
